# The identification and treatment of alcohol problems in primary care (iTAPP) study: protocol for a stepped wedge cluster randomized control trial testing *the 15-method* in a primary care setting

**DOI:** 10.1186/s13722-024-00474-6

**Published:** 2024-06-13

**Authors:** Peter Næsborg Schøler, Kristina Hasselbalch Volke, Sven Andréasson, Sanne Rasmussen, Jens Søndergaard, Anette Søgaard Nielsen

**Affiliations:** 1 The Unit for Clinical Alcohol Research, Department of Clinical Research, Faculty of Health Sciences, The University of Southern Denmark, Odense, Denmark; 2 The Research Unit for General Practice in Odense and Esbjerg, Department of Public Health, Faculty of Health Sciences, The University of Southern Denmark, Odense, Denmark; 3 Department of Mental Health Odense, Region of Southern Denmark, Odense, Denmark; 4 Department of Global Public Health, Karolinska Institutet, Stockholm, Sweden; 5 Brain Research - Inter-Disciplinary Guided Excellence, BRIDGE, University of Southern Denmark, Odense, Denmark

**Keywords:** Alcohol use disorder, Alcohol-related disorders, Primary health care, Screening and brief intervention, Randomized controlled trial

## Abstract

**Background:**

The 15-method is a targeted screening and treatment approach for alcohol problems in primary care. The 15-method used in primary care has proven as effective as specialized treatment for mild to moderate alcohol dependence in Sweden. A feasibility study of the 15-method in Danish primary care found the method acceptable and feasible.

**Aims:**

To evaluate the effectiveness of the 15-method in a Danish primary care setting in (1) lowering the proportion of patients exceeding the Danish low-risk alcohol consumption limit of ten standard units per week and a maximum of four standard units on a single day for men and women, and (2) increasing the likelihood of alcohol use being addressed during a consultation in general practice. Further, the rate of prescribed pharmacological treatment for alcohol problems (Disulfiram, Naltrexone, Acamprosate, and Nalmefene) will be measured along with the use of the biomarkers Alanine Transaminase and Gamma-Glutamyl Transferase.

**Methods:**

Stepped wedge cluster randomized controlled trial in sixteen general practices in the Region of Southern Denmark. Following a three-month baseline, the practices are randomly assigned to launch dates in one of four clusters. General practitioners and nurses receive three hours of training in the 15-method before launch. Patient questionnaires will collect data on alcohol consumption levels among patients affiliated with the practices. The healthcare professionals will register consultations in which alcohol is addressed in their patient filing system. Pharmacological treatment rates and the use of biomarkers will be collected through Danish national registries. The study follows the Medical Research Council’s guidelines for developing and evaluating complex interventions.

**Discussion:**

From the patient’s perspective, the 15-method may help identify alcohol-related problems at an earlier stage with flexible treatment offers in a familiar setting. For healthcare professionals, it addresses a traditionally challenging topic by equipping them with concrete tools, communication training, and clear treatment directives. From a societal perspective, primary care holds a unique position to identify hazardous and harmful alcohol use across different age groups, with potential public health and economic benefits through early identification and intervention.

**Trial registration:**

Clinicaltrials.gov NCT05916027. Retrospectively registered 22 June 2023.

**Supplementary Information:**

The online version contains supplementary material available at 10.1186/s13722-024-00474-6.

## Background

This study protocol reports on a randomized controlled trial evaluating the effectiveness of *the 15-method*, a novel intervention for identifying and treating alcohol problems in a primary care setting.

Excessive alcohol use is a large health risk factor [1] with significant economic costs globally [2]. The majority of alcohol problems are mild to moderate but also the least likely to receive treatment [3, 4]. The treatment gap has large negative public health consequences [5] and much is to be gained by treating alcohol problems at an earlier stage [6, 7]. Primary healthcare, specifically general practitioners (GPs), interacts with most of the population in the western world [8, 9], including persons with alcohol related problems [10]. However, most alcohol related problems are left untreated in primary care [11]. The patients may not view their alcohol consumption or themselves as needing specialized treatment, or they may wish to handle the problem themselves [12, 13]. Healthcare professionals (HCPs) may find it difficult to address potential alcohol problems [14], overlook problems that are not visible [15], or hold negative beliefs about substance use disorders and their treatment [16].

In Denmark, one in six exceed the national recommendations for weekly alcohol consumption [17] and five percent of yearly deaths are related to alcohol [5]. Despite national initiatives, only one in eight Danes with an Alcohol Use Disorder (AUD) seeks treatment [18].

Screening and brief intervention (SBI) for alcohol problems is an effective way to reduce alcohol consumption in hazardous and harmful drinkers in primary care [19] but it remains a challenge to implement brief interventions for alcohol problems [20, 21] mainly due to stigma [22, 23], shortness of resources [20, 24], policy [25, 26], and lack of tailored implementation strategies [21].

This has led to the development of a pragmatic intervention for alcohol problems designed for primary care: The 15-method [27]. The 15-method combines opportunistic screening and stepped-care treatment for alcohol problems and has proved to be a promising method for addressing and treating alcohol problems in primary care. Finn et al. demonstrated that the 15-method used in primary care was as effective as specialist care when treating patients with mild and moderate AUD [27, 28]. These findings support the notion that it is possible to treat individuals with mild and moderate AUD in general practice as effectively as in more intensive specialized treatment facilities [19, 29, 30].

A multistep evaluation and adaptation of the 15-method is underway in Denmark. The overall process follows the Medical Research Council (MRC) framework for developing and evaluating complex interventions [31]. As a first step, a feasibility study of the 15-method was conducted [32]. The feasibility study found that HCPs and patients consider the 15-method useful, and that the 15-method can be implemented in Danish general practice. The study also found that the 15-method needed contextual adjustments which have been completed and will be reported elsewhere. Following the MRC framework, the process has now moved to large scale evaluation to assess if the 15-method is recommendable for nationwide implementation.

The Identification and Treatment of Alcohol Problems in Primary Care (iTAPP*)* Study is a large-scale evaluation of the 15-method in Danish general practice and the second step of the overall multistep evaluation in Denmark.

### Aims

The iTAPP study will evaluate the 15-method’s effectiveness in:

1) Lowering the proportion of patients exceeding the Danish low-risk alcohol consumption limit of ten standard units per week and a maximum of four standard units on a single day for men and women.

2) Increasing the likelihood of alcohol use being addressed during a consultation in general practice.

### Methods and materials

This protocol adheres to the Standard Protocol Items: Recommendations for Interventional Trials (SPIRIT) Statement [33] (Supplementary File 1). Reporting on the intervention follows the Template for Intervention Description and Replication (TIDieR) Checklist [34] (Supplementary File 2).

### Trial design

The iTAPP study is designed as a stepped wedge cluster randomized controlled trial [35] in four steps. The study is pragmatic in its approach and designed to fit into a real-world clinical setting [36]. The randomization to clusters is conducted on practice level, as evaluation of interventions in general practices rarely offer feasible randomization on patient level [37]. Each cluster contributes to exposed and unexposed observations during the trial [35] and the design offers solutions to logistical considerations while supplying a framework for robust evaluation of the intervention [35, 38]. The study will combine qualitative and quantitative methodologies and include a process evaluation.

### Study setting

The iTAPP study is a collaborative study between The Unit for Clinical Alcohol Research at the Research Unit of Psychiatry Odense and The Research Unit of General Practice, Department of Public Health, The University of Southern Denmark.

The study will be conducted in general practices in the Region of Southern Denmark. All citizens with residence in Denmark are eligible for listing with a general practice, and 99% of Danish residents are affiliated with a GP [39, 40]. Consultation and treatment in general practice are free of charge. On average, all Danish citizens are in contact with their GP approximately seven times a year [41] and the GP is often the first point of contact to the health care system, as referral to e.g. office-based specialists and in- and outpatient hospitals occur through the GP in most cases. The GP is thus both first-line provider and gatekeeper to the secondary health care system [41]. Denmark has approximately 3500 GPs in 1650 practice units. The Region of Southern Denmark holds approximately 345 practice units [42] with an average of 2.3 GPs and 1,541 patients per GP, resulting in an average of 3,544 patients per general practice in the region. Practices are located in both urban and rural areas and include solo practitioners and company practices of up to 5 + GPs per practice [40]. The GPs own the practices and employ other HCPs, e.g., nurses, medical laboratory technicians, and assistants.

### Eligibility

All general practices in the Region of Southern Denmark will be eligible to participate in the study.

All clinic staff conducting patient consultations are eligible for inclusion, this including doctors, nurses, and other HCPs. Not all staff have to be enrolled in the study for the practice to be included. Each practice is allowed to decide whether all staff or only a selected group of staff will be participating in the study. All patients ≥ 18 years old affiliated with the participating practices are eligible.

### Exclusion and discontinuation criteria

Patients who are exempt from receiving digital post^1^ will be excluded.

Discontinuation criterium on practice level: Breach of contract between practice owner(s) and The University of Southern Denmark. Discontinuation criteria regarding the use of the intervention are not applicable as the intervention is (i) not monitored on patient or provider level, and (ii) not expected to worsen diseases.

### The intervention: the 15-method

The 15-method is an opportunistic SBI approach that interlinks basics from alcohol treatment and SBI research into a structured, stepped-care framework [43]. The name “*15-method*” denotes two things. The method targets patients who score more than 15 points on the Alcohol Use Disorder Identification Test (AUDIT) [44], indicative of alcohol dependence, and consultation are 15 min. The 15-method is based on Motivational Interviewing [45, 46], which is part of GP specialist training curriculum in Denmark, with home-work assignments based on basic elements from cognitive behavioral therapy [47] resulting in a patient-centered guided self-change [45, 46]. The 15-method differs from other SBI approaches in primary care by encompassing screening, assessment, and treatment in successive steps while offering flexible options in a familiar setting.

The first of the method’s three steps is an opportunistic screening in an already scheduled consultation. The method utilizes the variety of interactions in primary care to inquire about alcohol habits and HCPs can use the method during routine examinations or when discussing e.g. symptoms, clinical findings, or lab results. The HCP provides the patient with information on the findings in relation to alcohol consumption and offers brief advice with a menu of options [48, 49] including step two of the method. The patient completes the AUDIT questionnaire, which is suited for case-finding of alcohol problems in general practice [50, 51], prior to the second step.

The second step is a deeper assessment of possible connections between the patient’s alcohol use and findings from step one and can include a health check [52, 53]. The HCP and the patient focus on information relevant to the patient’s health situation and may include AUDIT scores, lab results, or specific symptoms. The HCP then provides brief personalized feedback and the next options are discussed through shared decision-making [54]. Options range from a follow-up to referral to specialist treatment and include progression to step three of the *15-method.*

The third step consist of up to three consultations of treatment via guided self-change [45, 46]. The HCP employs motivational interviewing during sessions and assigns homework based on cognitive behavioral therapy principles, which patients work on between sessions to promote self-change. Training in cognitive behavioral therapy is not required for HCPs to introduce these assignments. Each consultation and corresponding homework assignment holds a theme which includes self-monitoring, goal setting, identifying risk situations, and planning alternatives to drinking. The patient and HCP decide the treatment intensity and treatment goals through shared decision-making and treatment can include pharmacological aids (disulfiram, acamprosate, nalmefene, and naltrexone) following national guidelines. Through the process, the HCP and patient can revisit blood results, AUDIT scores, and other health indicators to track progress and maintain patient motivation. Treatment concludes with a follow-up for evaluation of the patient’s progress.

**Fig. 1:**
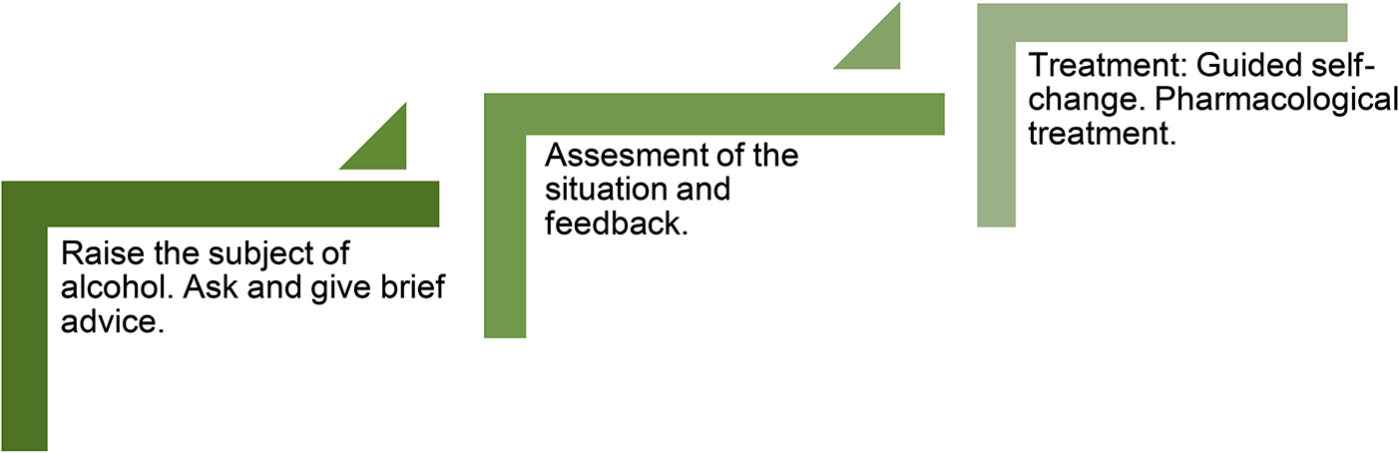
The three steps in the 15-method

Figure 1 presents the three steps in the 15-method and a detailed description of the method can be found in Supplementary File 3.

### Outcomes

1) Proportion of patients exceeding the Danish low-risk alcohol consumption limit of ten standard units per week and a maximum of four standard units on a single day for men and women.

2) The likelihood of alcohol use being addressed during a consultation in general practice.

3) Number of heavy drinking days (> 4 standard units on one day for men and women) per week among patients in the participating practices.

4) The use of the biomarkers Alanine Transaminase (ALAT) and Gamma-Glutamyl Transferase (GGT) in general practice.

5) Prescribed pharmacological treatment rate of Disulfiram, Naltrexone, Acamprosate and Nalmefene in general practice.

Table 1 presents an overview of study objectives, hypotheses, outcome measures, data sources, and data collection timepoints.

### Timeline

**Fig. 2:**
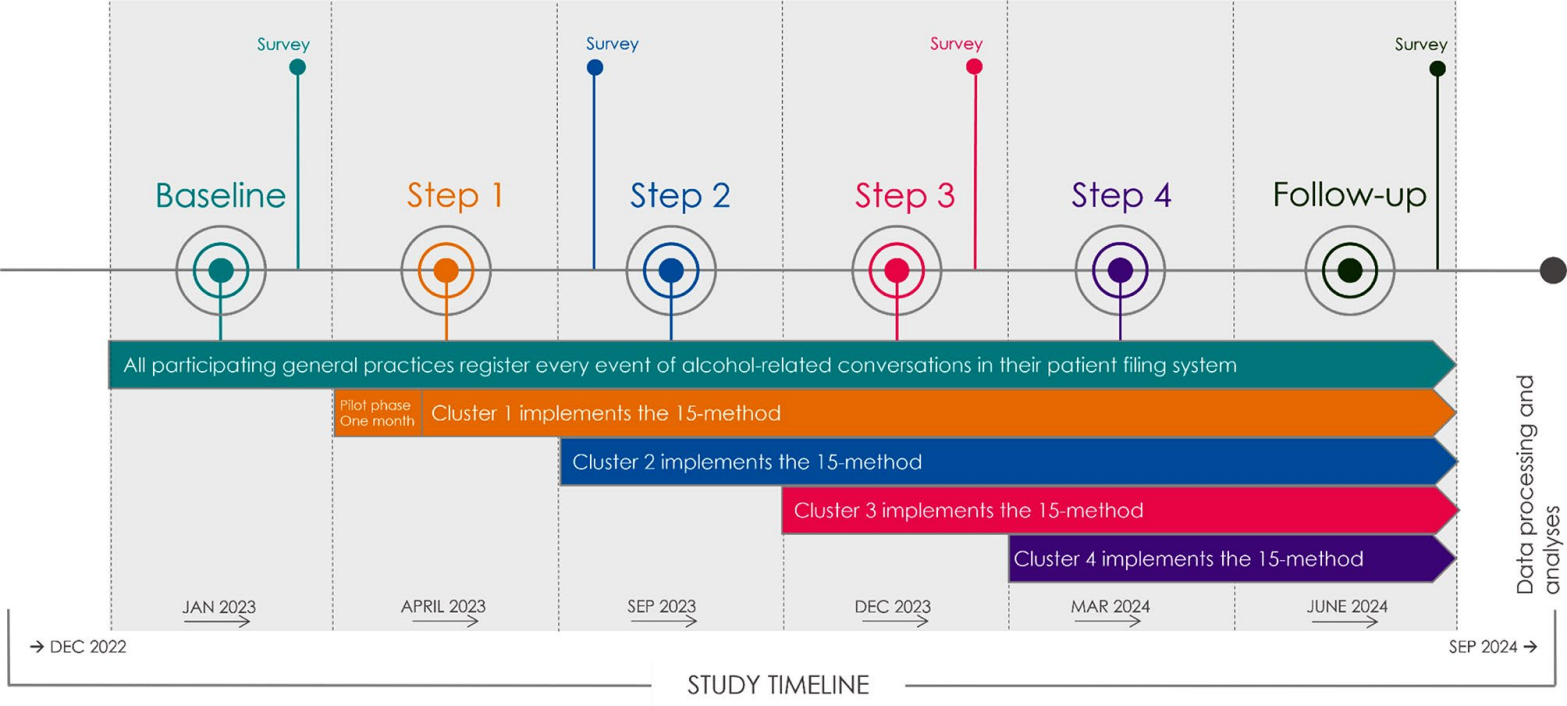
Timeline of the Identification and Treatment of Alcohol Problems in Primary Care (iTAPP) Study. Stepped wedge cluster randomized controlled trial design with general practices in The Region of Southern Denmark

Figure 2 presents the study timeline. The study will initiate with a three-month baseline period with no practices exposed to the intervention. During this period all participating practices are control-practices. Practices will be randomized in clusters of four and will cross from control group to intervention group, i.e. implementing the 15-method, in four steps during the intervention phase. A one-month pilot phase will be conducted as part of the first step for evaluation of implementation strategies. Following the pilot phase, each step will have a duration of three months. A three-month follow-up period will conclude the study period.

HCPs will register consultations in which alcohol is addressed from the start of the baseline period and throughout the study period (described below). Patient questionnaires will be sent four times during the study period; once during baseline, twice during implementation, and once at the end of the follow-up period.

### Implementation strategy

#### Introduction and baseline activities

The study will launch with a 2-hour introduction meeting for all participating practices. The meeting will inform on study timeline, organization, and practicalities, e.g., economic compensation and communication. HCPs who are unable to participate will be offered a one-hour online introduction. The research group will demonstrate how to register alcohol related consultations in the patient filing systems and instruct all HCPs to begin the registration (outcome measure 3).

During the baseline period, the participating practices will receive newsletters via e-mail. The newsletters will include reminders to continue the registration procedure of alcohol related consultations and information on the results from the randomization procedure. All practices will be offered open online Q-and-A sessions every three months.

All usual care and concomitant initiatives are allowed during the iTAPP study in the practices.

#### Active practices

The practices will be able to choose a three-hour timeslot accommodating their schedule for the 15-method training session. Training will be conducted at the practice facilities. Following the training session, the HCPs will have access to the study homepage which includes timeline, 15-method instruction videos and material, and a frequently asked questions section. The research team will provide nudging elements to the practice, e.g., mugs, flowers, and posters, as reminders to use the method and registration code. The research group will provide phone, e-mail, and video support as needed to address ad hoc challenges and questions and provide a separate e-mail newsletter to the active clinics. Practices will be trained in the two weeks leading up to the respective cluster launch date. Details on HCP training can be found in supplementary file S3.

#### Pilot phase

The initial month of step one will serve as a pilot phase (see Fig. 2) and focus on evaluating the implementation strategy and address any major unforeseen challenges. Interviews with HCPs from cluster one will be conducted, and the research group will discuss any relevant changes with the advisory board. No changes will be made to the 15-method itself or the allocated time for the training sessions.

#### Process evaluation

A process evaluation will be conducted following the MRCs guideline on evaluating complex interventions [55]. Interviews will be conducted in active practices once during step one (with cluster one), and once during step two (with cluster one and two). Interviews will include practice owner(s) and staff to ensure multilevel evaluation. The interviews will include a minimum of 20 HCP in both individual interviews and focus groups and will have a duration of thirty to sixty minutes. Data collection will be guided by the Consolidated Framework for Implementation Research (CFIR) [56]. The evaluation will seek to identify barriers and facilitators of implementation, adoption of the 15-method, and context elements impacting implementation outcomes [31, 57]. Reporting on any potential adaptations and modifications to the 15-method during the study will be done by The Framework for Reporting on Adaptations and Modifications to Evidence-based interventions (FRAME) [58]. Further, the interviews will be used to assess potential adverse events, unintended trial effects, intervention adherence, and trial conduct.

The evaluation will further assess fidelity to the 15-method in three ways. First, the interviews conducted via the CFIR interview guide include assessment of the implementation process and use of the intervention. Second, the HCP will be offered feedback on their conversational skills via audio recordings. If the HCP choose to, they can record consultations for upload to a secure server on which the conversations will be coded by KHV who is an experienced Motivational Interviewing Treatment Integrity (MITI) [59] coder and a member of the Motivational Interviewing Network of Trainers (MINT). The HCP will then receive feedback on the conversation and the collected data can be used as complementary fidelity assessment. Third, assessment of the use of the intervention will be evaluated through an electronic patient questionnaire on tablets in a small subset of randomly selected practices. Patients who just exited from a consultation will be asked to fill in a two-item questionnaire, stating whether the HCP inquired about alcohol habits in today’s consultation. The collected data will help qualify outcome measure three, i.e. the likelihood of alcohol use being addressed during a consultation in general practice.

### Data collection

#### Frequency of alcohol related consultations in the practices

The HCPs will register any consultation in which the topic of alcohol is raised using the International Classification of Disease tenth revision (ICD-10) code Z00.6 (Encounter for examination for normal comparison and control in clinical research program) [60] as secondary diagnosis to the consultations main diagnosis. In the case of no primary diagnosis to the consultation, the HCPs will use the International Classification of Primary Care 2 (ICPC-2) code A97 (no disease) [61, 62] together with the ICD-10 Z00.6 code. The HCPs will use the codes from start of baseline and throughout the study period. Any consultation in which alcohol consumption and/or alcohol-related problems is addressed will count as an observation regardless of whether the 15-method has been used.

Further, data on smoking, exercise, alcohol, and weight will be collected along with the total number of face-to-face consultations (including video consultations) in the practices from baseline to end of study. Local registration codes are available, among others, on smoking status (daily, occasionally, quit, never), exercise (hours per week), alcohol intake (standard units per week), and weight in all Danish general practices [63, 64].

Data will be collected on practice level only. A data quality assessment will be conducted during step two (Fig. 1) in a sub-sample of practices.

#### Questionnaire data on alcohol consumption, quality of life, and lifestyle

To measure the proportion of patients exceeding the Danish low-risk alcohol consumption limits a questionnaire will be sent to all patients ≥ 18 years affiliated to the participating general practices four times during the study period. The questionnaire will be sent by e-Boks [65] which is the official, digital communication route between public authorities and Danish citizens. The e-Boks system is accessed by the two-phased password system NemId/MitID [66], which is linked to the personal social security number of all residents in Denmark. A form ensuring informed consent from the participant will be attached to the questionnaire. To participate, and provide informed consent, the participant must press a link embedded in the form. The participant is informed that pressing the link will constitute as informed consent and the participant will be directed to the questionnaire. Questionnaire data will be stored via the secure Research Electronic Data Capture (REDCap) database [67] provided by Open Patient data Explorative Network (OPEN) [68]. Non-responders will receive a reminder after 7–10 days.

The questionnaire is adapted from the TAP-study [27] and the Relay-study [69] and assesses:

1. Alcohol consumption by (i) three questions on consumption from the Alcohol Use Disorder Identification Test (AUDIT-C) [44, 70], and (ii) daily alcohol intake within the last week by The Timeline Follow Back (TLFB) [71] one-week version. The TLFB one-week version has been successfully applied online and through questionnaires [72–74] and the AUDIT-C is validated in primary care [75].

2. Smoking, nutrition and exercise habits.

3. Health related quality of life by the EuroQol 5D (EQ-5D) questionnaire [76] which is validated worldwide [77, 78].

4. Current subjective mental well-being by the World Health Organization Five-Well-Being Index (WHO-5), also validated worldwide [79].

5. Whether the patient has attempted to change any aspect of their lifestyle (smoking, exercise, alcohol, diet) within the last year.

The questionnaire is designed to assess lifestyle and health related quality of life in general and to avoid a focus entirely on alcohol use. Alcohol use disorders are associated with potential stigmatization [11, 22], and to avoid response bias the questions on alcohol are embedded in questions about diet, physical activity, smoking, and mental well-being and formulated in a non-offending manner.

One standard unit of alcohol is defined as 12 g of pure alcohol.

#### Pharmacological treatment and biomarkers

This will include prescriptions of Disulfiram, Naltrexone, Acamprosate and Nalmefene. Data on prescriptions will be collected from the Danish National Prescription Registry [80]. Data on the use of biomarkers will include Gamma-Glutamyltransferase (GGT) and Alanine-Aminotransferase (ALAT) and will be collected via the National Laboratory Database. Data will be collected on practice level.

#### Patient evaluations

Patient experiences with the 15-method will be evaluated in a qualitative follow-up study. We will recruit patients through HCPs who have used the 15-method in collaboration with their patients.

#### Data management, monitoring, and confidentiality

Data will be stored on secured servers provided by OPEN [68] which is part of the data infrastructure in The Region of Southern Denmark. Questionnaire data will be collected using the REDCap database [67] provided by and stored at OPEN. Data management and monitoring will be provided via OPEN data managers. The research team will conduct regular quality control of the data in collaboration with OPEN data managers and have full access to the final trial dataset. No data monitoring committee will be established as (i) no risk to the participants is expected, and (ii) data will be anonymized and analyzed on population or clinic level. Data will be stored at OPEN servers after the study is concluded and can be transferred to The Danish National Archive for long term storage if deemed relevant.

### Procedures

#### Power and sample size calculation

We hypothesize that the implementation of the 15-method can lead to a 10% reduction in the proportion of patients exceeding the Danish low-risk alcohol consumption limits of ten standard units per week and a maximum of 4 standard units per drinking day for women and men, in the practices participating in the study. In the Region of Southern Denmark, the proportion of adults exceeding the national alcohol consumption limits is 14.2% [81]. We further hypothesize that the implementation of the 15-method will increase the proportion of consultations in which the topic of alcohol is addressed from 5.3 to 9.8% [82] in the participating practices.

Calculations follow power and sample size calculations in stepped wedge cluster randomized trials [83, 84], resulting in a total of 16 general practices being randomized to the 15-method in four steps of four practices (clusters). A power calculation was conducted for each of the two objectives, i.e., lowering the proportion of patients exceeding the low-risk alcohol consumption limit, and increasing the likelihood of alcohol use being addressed during a consultation. Sample size estimates were based on the most conservative result of the two. We expected an average of 1538 patients per GP in the region [85], 2.3 GPs per practice [85], 89% of patients within the targeted age-group (≥ 18 years) per general practice [86], and an estimated response rate in the patient questionnaires of 50% [87–89]. To detect the difference between the proportions 14.2% and 12.8% (10% reduction) using an exact binomial test of proportions with a 2.5% significance level and with an expected intraclass correlation coefficient of 0.068 [90], an estimated power of > 90% was obtained.

With a total of 16 general practices, a power of 81% can be achieved with a patient questionnaire response rate of 37%.

#### Recruitment and allocation

A computer-generated random sample of one hundred practices in The Region of Southern Denmark will be sent a letter of invitation to participate in the study. Non-responders will receive a follow-up phone call. Practices who show interest in participating will receive a formal visits or phone call to inform on the study. Practices owners will receive a written consent form including a contract with The University of Southern Denmark to be signed before enrolment. The recruitment procedure can be repeated if necessary.

Practices will be allocated to clusters based on a computer-generated random number sequence. As blinding to the intervention is not possible, information on the allocation sequence, i.e., which cluster the practice will belong to, will be distributed simultaneously to all practices via e-mail.

#### Test of patient questionnaire

The research group comprised a draft patient questionnaire from three validated questionnaires (AUDIT-C, EQ-5D, WHO-5), an alcohol consumption measure (TLFB one week version) and questions on smoking, nutrition, exercise, and efforts regarding habit change. The research group evaluated the content validity of the comprised questionnaire together with healthcare professionals and researchers from the Unit for Clinical Alcohol Research. Based on the feedback, the research group edited the draft and invited a second group to evaluate the updated version in a qualitative pilot test following the COSMIN checklist [91]. The second test group consisted of researchers from The Research Unit of General Practice Odense with expertise in questionnaire development, questionnaire-based research and patient reported outcome measures. Evaluations from the pilot testing were used to finalize the patient questionnaire.

### Analysis plan

#### Quantitative analyses

Analyses will be conducted by intention-to-treat principle [92]. Binary outcomes will be analyzed using mixed model logistic regression. Continuous outcomes, e.g., number of heavy drinking days, the use of biomarkers, and pharmacological prescription frequency will be analyzed using mixed model linear regression. Outcomes will be analyzed on practice level with weighted observations according to patient population size and number of participating HCPs in the practice [93].

#### Qualitative analyses

The CFIR interview guide [56] will constitute the basis of the coding framework. The CFIR guide (cfirguide.org) supplies a pre-populated coding template that will be utilized for coding. The deductive approach [94] of using the preset coding tree from the CFIR template will be complimented by abductive analysis [95, 96] to allow for unexpected findings and theoretical considerations to the identified determinants. The CFIR framework can describe contextual factors and help generate hypotheses for implementation success or failure, but it does not adhere to a single theory to explain actions behind the effects of the determinants [56, 97]. By allowing for potential new insights via an abductive approach, considerations to the theoretical underpinnings behind the identified determinants becomes possible. Interviews will be transcribed verbatim by PNS and a research assistant and PNS will conduct the initial coding. Double coding will be conducted on a sub-set of data for reliability measures and discrepancies or disagreements will be discussed within the research team. Practices will be created as cases based on their characteristics and CFIR constructs will be rated across cases. Cases will then be analyzed across CFIR constructs using Nvivo framework matrices and case classification supplied by hierarchy charts. The research team will discuss data saturation [98] and information power [99] to ensure adequate levels of collected data.

The Framework for Reporting Adaptations and Modifications to Evidence-Based Interventions (FRAME) [58] will be utilized to facilitate a better understanding of local adaptations to the 15-method and differences in the implementation processes in the practices. The FRAME focuses on describing (1) when modifications were made, (2) whether the modification was plan or unplanned, (3) who participated in the decisions about modification, (4) what was modified, (5) level of delivery of the modified intervention, (6) modifications in relation to intervention fidelity, and (7) the rationale for the modification(s) [58]. The analysis of FRAME components will be informed by the CFIR cross-case analyses described above.

Analyses will be conducted using Nvivo 12 [100] or newer.

### Trial status spring 2024

As of March 2024, the iTAPP study includes eighteen practices. The first three clusters are active and use the 15-method in their everyday practice. The process evaluation is underway and currently concluding its data collection phase. Three of the four patient questionnaires have been distributed and yielded an average response rate of 30%. Three practices from the Danish feasibility study of the 15-method in primary care have continued as participants in the iTAPP study. As these three practices were familiar with the 15-method prior to the iTAPP study they are not included among the eighteen practices and data analyses will be kept separate.

## Discussion

The iTAPP study is pragmatic in its approach [101] and the implementation strategy focuses on a flexible engagement of practice staff and managers (GPs). This is done by offering the same training and support to all levels of healthcare professionals (nurses, GPs, assistants) while encouraging them to use the method as they see it best in their practice. The reason for this strategy is three-fold. First, many GPs decline to participate in projects due to time constraints [102]. We address this initial barrier by making the participation as flexible as possible. Second, results from the feasibility study of the 15-method in Denmark indicated that the GPs wanted different levels of involvement with the 15-method intervention and wanted the opportunity to delegate tasks to their staff [32]. Third, results from the feasibility study also indicated that the 15-method was well suited for interdisciplinary collaboration, which improved the feasibility and use of the method.

This study offers a multifaceted perspective, with four key viewpoints. From the patient’s perspective, the 15-method can potentially help identify alcohol related problems at an earlier stage, and provide a flexible treatment offer in a familiar setting. For healthcare professionals, the 15-method addresses a traditionally challenging topic [11, 14] by equipping them with concrete tools, communication training, and clear treatment directives. From a societal perspective, the accessibility of primary care positions GPs uniquely to identify hazardous and harmful alcohol use across diverse age groups, with potential public health and economic benefits through early identification and intervention. Should the iTAPP study substantiate the effectiveness of 15-method in reducing alcohol consumption within a primary care setting, it can form the basis for national roll-out strategies. Finally, it may prompt deliberations regarding the responsibilities and economic incentivization of Danish general practitioners concerning the management of alcohol related problems.

### Methodological considerations

The level of alcohol related consultations is measured in the same way throughout the study. The HCPs are asked to register any consultation in which alcohol is addressed from start of baseline. The knowledge of being observed in this way can potentially introduce a Hawthorne effect prior to intervention implementation. Although the actual impact of the Hawthorne effect is debated [103] the increased focus on registering consultations can potentially decrease the detectable difference to post implementation levels. Given the sample size we do however expect these potential differences to even out.

The 15-method training will be conducted in three-hour sessions, shorter than the original Swedish training. Results from the Danish feasibility study suggested that the Swedish material was too extensive for Danish general practice and that training could be condensed via shortening of the material. Moreover, most aspects of the 15-method are already familiar to HCPs, as they are accustomed to concepts such as MI, patient homework assignments, and opportunistic symptom-based screening. Therefore, the training will serve as a review of familiar concepts while providing a comprehensive introduction to the 15-method to integrate knowledge effectively.

The patient questionnaires will be distributed with consideration to known seasonal variations in alcohol consumption, e.g., holiday seasons. People with low IT-literacy, e.g., elderly and cognitive impaired can opt-out of the electronic mail system through which the questionnaire is distributed. As a results of this, a small patient sample will not be available for collection of questionnaire data. Further, as the questionnaire is distributed four times during the study period, we anticipate some patients will choose not to answer the subsequent questionnaires.

The randomization procedure is conducted on practice level. Randomization on patient level is not possible as the study investigates the effectiveness of the intervention on the initial screening procedure among non-selected patients. Further, a randomization by HCPs would increase the risk of spillover effect within the practice.

The study’s stepped wedge design is a strength as it allows for a brief pilot phase, for continuous evaluations of resources throughout the study, and for logistical considerations regarding implementation and support. The design follows the MRC framework for complex interventions and builds on feasibility testing [32] and pre-trial adjustments to the intervention. Another strength is the imbedded process evaluation which can help describe relationships between context, mechanisms of impact, and study outcomes.

**Table 1:** Objectives, hypotheses, outcome measures, data sources and data collection timepoints in the Identification and Treatment of Alcohol Problems in Primary Care (iTAPP) Study. Stepped wedge cluster randomized controlled trial design with general practices in The Region of Southern Denmark

Objective	Hypothesis after implementation of the 15-method:	Outcome measure	Data source	Data collection timepoint
i) To evaluate the 15-method’s effectiveness in lowering the proportion of patients who exceed the Danish low-risk alcohol consumption levels.	The proportion of patients exceeding the Danish national low-risk alcohol consumption limits will be reduced.	Proportion of patients exceeding the Danish low-risk alcohol consumption limit of ten standard units per week and a maximum of four standard units on a single day for men and women.	Questionnaires to all patients 18 + years affiliated with participating practices including the AUDIT-C, and TLFB one week version.	Four times during trial. Illustrated in Fig. 2.
ii) To evaluate the 15-method’s effectiveness in increasing the likelihood of alcohol use being addressed during a consultation in general practice.	Alcohol related consultations will occur more frequently in the general practices.	The likelihood of alcohol use being addressed during a consultation in general practice.	Registration codes from practice patient filing systems: ICPC-2 code A97, and the ICD-10 code z006.	Once during the trial period (data quality control). Once at end of follow-up period.
iii) To evaluate the 15-method’s effectiveness in lowering the number of heavy drinking days per week in patients.	The number of heavy drinking days will decrease in the patient population.	Number of heavy drinking days (> 4 standard units on one day for men and women) per week in patients.	Questionnaires to all patients 18 + years affiliated with participating practices including the AUDIT-C, and TLFB one week version.	Four times during trial. Illustrated in Fig. 2.
iv) Evaluate the frequency of use of biomarkers as a screening tool for harmful alcohol use.	The use of biomarkers as a screening tool for harmful alcohol use will increase.	The use of the biomarkers ALAT and GGT.	The Danish National Laboratory Database.	End of follow-up period.
v) Evaluate the prescription rate of pharmacological treatment for alcohol problems.	Prescriptions of pharmacological treatment for alcohol problems will increase, as the HCPs become more aware of use cases and more familiar with treatment options through academic detailing.	Prescription rate of Disulfiram, Naltrexone, Acamprosate and Nalmefene.	The Danish National Prescription Registry.	End of follow-up period.

*Notes* One standard unit defined as 12 g of ethanol; AUDIT-C, Alcohol Use Disorder Identification Test Consumption; TLFB, Timeline Follow Back; EQ-5D, European Quality of Life 5 Dimensions; WHO-5, World Health Organization Five Well Being Index; ICPC-2, International Classification of Primary Care 2; ICD-10, International Classification of Disease tenth revision; code A97, no disease; code z006, Encounter for examination for normal comparison and control in clinical research program; ALAT, Alanine Transaminase; GGT, Gamma-Glutamyl Transferase; HCP, health care professional

### Electronic supplementary material

Below is the link to the electronic supplementary material.

Supplementary Material 1

Supplementary Material 2

Supplementary Material 3

## Data Availability

Not applicable. Project materials can be accessed at The iTAPP study homepage www.sdu.dk/en/itapp-study and the Danish 15-method material can be accessed at www.sdu.dk/en/15-metoden www.sdu.dk/en/15-metoden

## Footnotes

1 In 2021 a total of 338.755 citizens, equivalent to 6,9% of the adult Danish population, were exempted from receiving Digital Post (digst.dk/it-loesninger/digital-post/om-loesningen/tal-og-statistik/). To be exempt from receiving digital post, a citizen must be unable to receive Digital Post and must meet at least one exemption criterium. The exemption criteria are set by the Danish Agency for Digital Government and include the following: 1) Suffering from a physical or mental disability that prevents you from using Digital Post, 2) Not having access to a computer, smartphone or tablet in your home or place of residence, 3) Having left Denmark permanently, 4) Not being registered with a permanent address or place of residence in Denmark, 5) Having limited command of Danish, which makes it difficult to use Digital Post, 6) Having practical difficulties in obtaining the electronic ID giving access to Digital Post, 7) Living in an area without Internet connection, 8) Other special circumstances deemed relevant by the Agency for Digital Government https://www.retsinformation.dk/eli/lta/2021/2017/ The citizen is required to apply for exemption in person at the local municipality.

## Declarations

### Protocol amendments

Important modifications and amendments to the protocol will be reported to trial participants, trial registries, and journals.

### Dissemination policy

Results will be presented at national and international scientific events and published along with the study protocol in scientific peer reviewed journals. Authorship will follow the International Committee of Medical Journal Editors guidelines.

## Abbreviations

HCP: Health Care Professional
AUD: Alcohol Use Disorder
MRC: Medical Research Counsil
GP: General Practitioner
SPIRIT: Standard Protocol Items: Recommendations for Interventional Trials
TIDieR: Template for Intervention Description and Replication
AUDIT: Alcohol Use Disorder Identification Test
AT: Alanine Transaminase
GGT: Gamma-Glutamyl Transferase
CFIR: Consolidated Framework for Implementation Research
FRAME: Framework for Reporting on Adaptations and Modifications to Evidence-based interventions
ICD-10: International Classification of Disease tenth revision
ICPC-2: International Classification of Primary Care 2
REDCap: Research Electronic Data Capture
OPEN: Odense Patient data Explorative Network
COSMIN: COnsensus-based Standards for the selection of health status Measurement Instruments
