# Supplementary material for: The identification and treatment of alcohol problems in primary care (iTAPP) study: protocol for a stepped wedge cluster randomized control trial testing the 15-method in a primary care setting
Source: Addict Sci Clin Pract. 2024 Jun 13;19:49. doi: 10.1186/s13722-024-00474-6 (PMC11170864; doi:10.1186/s13722-024-00474-6)
Supplement: Supplementary file 3 — Supplementary Material 3 [file 13722_2024_474_MOESM3_ESM.docx]

# Detailed description of the intervention: The 15-method

## The three steps of the 15-method

*Step one: Opportunistic screening followed by brief advice.* The opportunistic screening is a clinical assessment of relevant opportunities to address a certain topic. In the case of the 15-method, the opportunistic screening is a screening for alcohol related problems when deemed relevant by the healthcare professional (HCP). Relevant symptoms include somatic and mental health symptoms that may be related to or worsened by alcohol intake (1, 2). Step one thus involves an increased attention of risky alcohol use or alcohol-related health concerns and focuses on addressing the topic of alcohol *in relation* to the patient’s symptoms or reason for contacting the HCP. If the topic of alcohol habits is considered relevant by the HCP and/or the patients express concerns about their alcohol use, moving on to step two is suggested. The HCP can elaborate the screening by an objective assessment of the patient’s alcohol use using the Alcohol Use Disorder Identification Test (AUDIT) which is suited for identification of alcohol problems in general practice (3, 4). Screening can further include clinical examinations and use of the biological markers Alanine Transaminase (ALAT) and Gamma-Glutamyl Transferase (GGT). The HCP can then recommend the patient to fill in the AUDIT questionnaire before the next consultation, which can serve as link between step one and step two.

Step one is not an independent consultation in addition to the planned consultation. Rather, step one is performed as an integrated part of an already planned consultation, with the option of scheduling an additional consultation if relevant (step 2).

*Step two: Assessment based on patient self-reported AUDIT scores and/or biological markers.* The HCP offers the patient feedback based on the reported AUDIT scores as well as information on the potential consequences of the patient’s alcohol consumption (5, 6). This session is a possibility to assess potential alcohol problems in depth and through professional feedback to enhance the patient’s motivation to change. The HCP assists the patient in reflecting on their alcohol habits and encourages the patient to set a goal for reducing alcohol intake. The HCP can offer a follow-up consultation in two to four weeks depending on the situation and patient’s needs. If step two is not sufficient, and the patient is interested in further support to reduce their alcohol intake, the HCP can suggest moving to step three. The healthcare professional can utilize additional questionnaires depending on the patient situation which include: The Timeline Follow-Back (7) one-week version; The Short Alcohol Dependence Data Questionnaire (8); a questionnaire on alcohol habits based on the International Classification of Diseases version 10 (ICD-10) criteria for alcohol dependence (9); a questionnaire on the use of other addictive substances (nicotine, narcotics, and benzodiazepines or opioids). The patient can fill in these questionnaires as a link between step two and step three.

*Step three: Treatment based on Motivational Interviewing and Guided Self-Change (10, 11)*. This step consists of up to three treatment sessions and a follow-up. The HCP can provide adjuvant material, which include an alcohol logbook (diary style notebook) and homework assignments. The treatment intensity and goal setting is based on shared decision-making (12). Pharmacological treatment (Disulfiram, Acamprosate, Nalmefene, or Naltrexone) is a concomitant treatment option and follows national guidelines. Each session and affiliated homework assignment include a theme to facilitate behavior change, such as goal setting, self-monitoring of alcohol consumption, identification of risk situations or action plans for alternatives to drinking.

## Intervention Material

Intervention material for HCPs and patients has been adjusted to Danish general practice based on results from a feasibility study of the 15-method in Danish general practice (13). The Danish version of the 15-method includes the following material:

- A manual for HCPs describing the content and structure of the 15-method.
- An index map (quick guide) for HCPs, summarizing the structure and key components of the 15-method.
- A logbook for patients containing logbook pages for recording of daily alcohol consumption and other lifestyle factors and homework assignments, reflection pages, motivational quotes, and appointment reminders.
- Printed homework assignments to be used as stand-alone reflections (identical to the assignments included in the logbook). Intended for patient who prefer less material or where the complete patient logbook is not relevant.
- Printed AUDIT questionnaires ready for use by the HCPs as part of the assessment of the patients’ alcohol use.
- Posters and flyers for use in the participating practices, e.g. in the waiting room, encouraging patients to talk about their alcohol habits with their HCP.
- Nudging elements for the HCPs, e.g. mugs and bottles bearing the message “let’s talk about it” (alcohol habits) with suggestions on who to talk to.

All material (with the exception of posters, flyers, and nudging elements) can be accessed at [www.sdu.dk/en/15-metoden](http://www.sdu.dk/en/15-metoden) (material in Danish).

## Training in the 15-method

Training of the HCPs will be done through academic detailing, also referred to as educational outreach visits (14, 15). Academic detailing is described as “a personal visit by a trained person to health professionals in their own setting” and has shown to be an effective approach for changing clinicians’ practice (14). The aim of academic detailing is to provide unbiased information for the professionals with the intention of changing their clinical practice, enhancing evidence-based care and improving patient outcomes (16).

The training will be carried out by PNS and KHV at the practice units. PNS is a medical doctor and PhD-student in the field of clinical alcohol research and treatment, and KHV has a MSc in Social Work and solid clinical experience from the specialized addiction treatment services. The training will consist of a three-hour session, divided into three parts. The first part will include a reflection on and definition of alcohol problems, both within a clinical and cultural context. Further, a brief overview of the alcohol consumption levels in Denmark, along with demographics and characteristics of citizens with a risky drinking pattern. Emphasis will be on the clinical presentation and recognition of symptoms potentially related to or affected by alcohol intake. The HCP are familiar with most of such symptoms through their professional training and the session will focus on a brush-up and to fill any identified knowledge gaps. Part two will consist of a thorough introduction to the 15-method material and structure and will constitute most of the training session. The training will also focus on a motivational approach, including awareness on stigma, and how to address the topic of alcohol. General practitioners in Denmark are trained in motivational interviewing through their specialist training, and most nurses in Danish general practice are familiar with the approach, as they often are responsible for life-style related consultations (e.g. smoking cessation). Thus, this part of the 15-method training is not an introductory course on motivational interviewing but a re-introduction or recapitulation of the MI approach with concrete examples for use in alcohol related consultations. Part three will focus on practical information regarding the iTAPP Study, such as study timeline, introduction to homepage and instruction videos, and available support options.

## Applying the 15-method in clinical practice

After completing the training, the HCPs are encouraged to apply the 15-method in their daily practice. The HCPs will be able to apply the 15-method instantly in their clinical practice, as step 1 of the method consists of an increased attention to alcohol and an opportunistic screening for alcohol related problems in already scheduled consultations.

# References

1. Coulton S, Drummond C, James D, Godfrey C, Bland JM, Parrott S, et al. Opportunistic screening for alcohol use disorders in primary care: comparative study. BMJ. 2006;332(7540)

2. Kaner E, Bland M, Cassidy P, Coulton S, Dale V, Deluca P, et al. Effectiveness of screening and brief alcohol intervention in primary care (SIPS trial): pragmatic cluster randomised controlled trial. BMJ. 2013;346

3. Allen JP, Litten RZ, Fertig JB, Babor T. A review of research on the Alcohol Use Disorders Identification Test (AUDIT). Alcohol Clin Exp Res. 1997;21(4)

4. Reinert DF, Allen JP. The Alcohol Use Disorders Identification Test (AUDIT): a review of recent research. Alcohol Clin Exp Res. 2002;26(2)

5. Chick J, Ritson B, Connaughton J, Stewart A, Chick J. Advice versus extended treatment for alcoholism: a controlled study. British Journal of Addiction. 1988;83(2)

6. Miller WR, Sovereign RG, Krege B. Motivational interviewing with problem drinkers: II. The Drinker's Check-up as a preventive intervention. Behavioural and Cognitive Psychotherapy. 1988;16(4)

7. Sobell LC, Sobell MB. Timeline follow-back. Measuring alcohol consumption: Springer; 1992. p. 41-72.

8. Raistrick D, Dunbar G, Davidson R. Development of a questionnaire to measure alcohol dependence. British journal of addiction. 1983;78(1)

9. World Health Organization. The ICD-10 classification of mental and behavioural disorders: diagnostic criteria for research: World Health Organization; 1993.

10. Sobell MB, Sobell LC. Problem drinkers: Guided self-change treatment: The Guilford Press; 1996.

11. Andréasson S, Hansagi H, Österlund B. Short-term treatment for alcohol-related problems: four-session guided self-change versus one session of advice—a randomized, controlled trial. Alcohol. 2002;28(1)

12. Elwyn G, Frosch D, Thomson R, Joseph-Williams N, Lloyd A, Kinnersley P, et al. Shared decision making: a model for clinical practice. Journal of general internal medicine. 2012;27(10)

13. Schøler PN, Søndergaard J, Barfod S, Nielsen AS. Danish feasibility study of a new innovation for treating alcohol disorders in primary care: the 15-method. BMC Primary Care. 2022;23(1)

14. O'Brien MA, Rogers S, Jamtvedt G, Oxman AD, Odgaard-Jensen J, Kristoffersen DT, et al. Educational outreach visits: effects on professional practice and health care outcomes. Cochrane Database Syst Rev. 2007;2007(4)

15. Van Hoof TJ, Harrison LG, Miller NE, Pappas MS, Fischer MA. Characteristics of Academic Detailing: Results of a Literature Review. Am Health Drug Benefits. 2015;8(8)

16. Yeh JS, Van Hoof TJ, Fischer MA. Key Features of Academic Detailing: Development of an Expert Consensus Using the Delphi Method. Am Health Drug Benefits. 2016;9(1)
